# Syphilis and the Eye: Clinical Features, Diagnostic Challenges, and Evolving Therapeutic Paradigms

**DOI:** 10.3390/pathogens14090852

**Published:** 2025-08-27

**Authors:** Zizhen Ye, Mingming Yang, Yaru Zou, Jing Zhang, Jiaxin Deng, Yuan Zong, Kyoko Ohno-Matsui, Koju Kamoi

**Affiliations:** 1 Department of Ophthalmology & Visual Science, Graduate School of Medical and Dental Sciences, Institute of Science Tokyo, Tokyo 113-8510, Japan; yezizhen518@gmail.com (Z.Y.); yangmm-12@outlook.com (M.Y.); alicezouyaru519@gmail.com (Y.Z.); zhangj.c@foxmail.com (J.Z.); dengjiaxin.med@gmail.com (J.D.); zongyuan666@gmail.com (Y.Z.); k.ohno.oph@tmd.ac.jp (K.O.-M.); 2 Department of Ophthalmology, Zhongshan Torch Development Zone People’s Hospital, Zhongshan 528436, China

**Keywords:** syphilis, ocular syphilis, uveitis, neurosyphilis, multimodal imaging, optical coherence tomography, Serologic tests, Penicillin G

## Abstract

Syphilis is a systemic infection with a broad spectrum of ocular involvement that can affect every segment of the eye. Clinical presentations range from interstitial keratitis, conjunctivitis, episcleritis, and scleritis to anterior, intermediate, and posterior uveitis; acute syphilitic posterior placoid chorioretinitis; retinitis; retinal vasculitis; neuroretinitis; optic neuritis; exudative retinal detachment; and optic nerve dysfunction. These manifestations may occur at any stage of infection and are frequently nonspecific, contributing to diagnostic delays. Diagnosis requires a high index of suspicion and is established by combined non-treponemal and treponemal serologic testing, with cerebrospinal fluid analysis when neurosyphilis is suspected. Multimodal imaging, including optical coherence tomography, fluorescein angiography, fundus autofluorescence, and visual field testing, enhances the detection of subclinical and atypical diseases. Management mandates prompt intravenous penicillin G, with adjunctive corticosteroids to mitigate Jarisch–Herxheimer reactions and control inflammation; ceftriaxone or doxycycline serve as alternatives for penicillin-allergic patients. Long-term follow-up with serial serologies and neurologic evaluation is essential to detect relapse or progression to neurosyphilis. Despite effective therapy, diagnostic delays contribute to irreversible visual loss in a significant proportion of cases. This review integrates current knowledge on ocular syphilis, emphasizing its varied presentations and the importance of early recognition to prevent vision-threatening complications, and calls for multidisciplinary, mechanism-based research to optimize outcomes. We conducted a literature search in Pubmed and Embase for articles published between 2000 and 2025, using the terms “ocular syphilis,” “syphilitic uveitis,” and “neurosyphilis,” with a focus on epidemiology, clinical features, diagnostics, therapeutics, and co-infections.

## 1. Introduction

Syphilis, one of the three sexually transmitted infections of major global importance, is caused by the spirochete *Treponema pallidum* [1,2]. Untreated syphilis, while curable, can lead to irreversible and potentially fatal complications that impact the neurological, cardiovascular, ocular, and hepatic systems, thus presenting a persistent global health challenge for over 150 years. Syphilis is responsible for over 300,000 fetal and neonatal deaths, with an additional 215,000 infants facing an elevated risk of early mortality [3]. However, since the onset of the 21st century, there has been a marked increase in the global prevalence of syphilis in both developed and developing nations [4]. In 2022, over 8 million individuals aged 18 to 49 were diagnosed with syphilis worldwide [5]. That same year, the United States reported its highest annual number of syphilis cases since 1950 [6].

Ocular structures are among the most frequently affected extra-genital sites in syphilis. According to findings from the British Ocular Syphilis Study (BOSS), which conducted nationwide surveillance over a two-year period, intraocular syphilis occurs at a rate of 0.3 cases per million adults annually [7]. However, this rate increases significantly in the presence of immunocompromised conditions, particularly in cases of HIV co-infection. Approximately one-third of HIV co-infected patients report ocular involvement. Among all individuals infected with syphilis, 2–3% reported ocular manifestations.

In response to the rising global burden of syphilis, the World Health Organization (WHO) announced a new strategy for combating sexually transmitted infections (STIs) for the period from 2022 to 2030. This strategy aims to eliminate STIs as a major public health threat by the end of the decade [2]. A key component of this plan is increasing awareness of syphilis among both healthcare professionals and the general population. As part of this broader effort, ocular syphilis—an often overlooked but potentially vision-threatening manifestation—requires particular attention. In this review, we summarize the latest developments in the understanding of ocular syphilis, including its clinical characteristics, diagnostic approaches, and recent advancements in ophthalmic imaging and treatment. The aim of this review is to provide a clinician-oriented synthesis prioritizing diagnostic pitfalls and imaging-driven management. We also discuss the impact of co-infections with HIV and human T-cell leukemia virus (HTLV) on disease presentation and prognosis.

### Epidemiology and Pathogenesis of Ocular Syphilis

The rising global prevalence of syphilis correlates with an increase in the incidence of ocular syphilis. Following a worldwide nadir in 2001, ocular syphilis has shown a steadily increasing trend [8]. In recent years, studies from various regions have reported a rise in ocular involvement, highlighting its re-emergence as a significant public health concern. A six-year study conducted in eastern China from 2016 to 2021 showed a year-on-year increase in ocular syphilis [9]. Another study from West Virginia assessed the prevalence of ocular syphilis, along with comorbidities, and visual outcomes over a decade. The incidence of systemic syphilis rose from 27 cases in 2010 to 105 cases in 2020, demonstrating a distinct upward trend in ocular syphilis cases [10]. More than 90% of reported ocular syphilis cases occur in males [7,11], suggesting a strong gender and behavioral predisposition. Notably, men who have sex with men (MSM) are estimated to be up to 250 times more likely to contract primary and secondary syphilis compared to women, further highlighting the elevated risk in this population [12]. It should be noted that many of the included studies are clinic-based and may be subject to ascertainment bias.

If left untreated, syphilis progresses through four distinct stages: primary, secondary, latent, and tertiary. Ocular manifestations can occur at any stage but are most commonly observed during the secondary and latent phases. Approximately 28% of ocular involvement is reported during the secondary stage [13,14,15]. Ocular syphilis is considered a type of neurotropic infection that can affect any part of the eye and present in different ways [16]. The clinical manifestations of ocular syphilis differ according to the stage of the disease. During the acute phase, characterized as occurring within six months of onset, common findings consist of vitreous haze, neuroretinitis, and acute posterior placoid chorioretinitis. During the chronic phase (≥6 months), patients frequently exhibit diffuse chorioretinitis, pseudoretinitis pigmentosa, and macular complications, including cystoid macular edema (CME) and the formation of epiretinal membranes [17].

Ocular syphilis occurs when *Treponema pallidum* penetrates the blood–ocular and blood–retinal barriers, facilitating colonization of immune-privileged areas. The spirochete expresses outer membrane proteins, including *Tp0751*, and exhibits antigenic variation in *TprK*, facilitating endothelial adhesion, tissue invasion, and immune evasion. The mechanisms enable persistence in the retina and choroid, while the host immune response, especially T-cell-mediated inflammation, exacerbates tissue damage and results in the varied clinical phenotypes observed [18,19].

## 2. Clinical Manifestation of Syphilis-Related Ocular Disease

### 2.1. Anterior Segment Manifestations

Anterior uveitis: Syphilitic uveitis is an ocular inflammation that has various and nonspecific manifestations. Syphilitic uveitis may present similarly to other infectious or autoimmune forms of uveitis [20], making its diagnosis particularly difficult and often requiring a considerable observation period. Patients usually present with symptoms like blurred vision, floaters, red eye with pain, or conjunctival injection [21]. Although in most reports posterior uveitis is predominant in syphilitic uveitis, some studies have shown a slight difference in distribution. Alhawsawi et al. recently found that anterior uveitis (25.9%) was the most common type in a group of people from Saudi Arabia. It was followed by posterior uveitis (22.4%) and panuveitis (20.7%). This finding illustrates how the disease can be mistaken for autoimmune diseases [15]. Shahid et al. also saw a similar distribution in a group of 89 affected eyes: 25.9% had anterior uveitis, 22.4% had posterior uveitis, and 18.8% had panuveitis [22]. This difference in the spectrum of syphilitic uveitis may be due to the difference in patient populations and immune status [23].

Individual case reports indicate that primary syphilis infrequently presents as the chancre of the eyelid, underscoring the diagnostic difficulties, particularly in patients with HIV. Cillino et al. described an HIV-positive male who initially presented with a painless eyelid chancre, misdiagnosed as a chalazion. The lesion appeared as a small, painless, resolving ulcer with slightly elevated margins located above the lateral canthus of the left eye [24]. Although cerebrospinal fluid analysis was unremarkable, serological testing confirmed syphilis reinfection [25].

Syphilitic Scleritis: Syphilitic scleritis is a rare but sight-threatening subtype of secondary syphilis. Painful, red eyes are the most common manifestation among those who have syphilitic scleritis [26]. Syphilitic scleritis should be considered in instances of severe anterior segment inflammation [27,28], especially when necrotizing scleritis is seen [28]. According to a study that included 44 cases of scleritis (50 eyes) and 9 cases of superficial scleritis (14 eyes), anterior scleritis is the most common type of syphilitic scleritis, with a prevalence of over 90% among syphilitic scleritis patients. Nodular anterior scleritis represents the most common subtype. (58.1%) [26]. A review of the literature on syphilitic scleritis revealed greater resemblance to non-infectious rather than infectious scleritis. Scleral necrosis, commonly seen in infectious scleritis, was present in only 16.1% of syphilitic cases—far lower than the >90% reported in other infectious scleritis series. Moreover, hallmark features of infectious scleritis, such as scleral ulcers with calcific plaques, microabscesses, and suppurative discharge, were absent in syphilitic cases [26].

Syphilitic scleritis is easy to overlook and often causes a diagnostic dilemma. Lesions in the anterior segment are mistaken for rheumatoid-related inflammation or viral conjunctivitis. The outlook for superficial scleritis is generally good, and it rarely recurs. However, it should not be ignored as a possible early sign of active neurosyphilis. A study found that four patients with uncomplicated ocular scleral changes later developed neurosyphilis and fully recovered after standard treatment. This shows that scleritis and superficial scleritis should be included in the screening process for neurosyphilis [29].

Interstitial keratitis: Interstitial keratitis (IK) with conjunctivitis is an uncommon manifestation of syphilis, considered an immune-mediated inflammatory reaction that was more common before the introduction of antibiotics for treatment [14,30]. However, it is considered the most characteristic and significant ocular symptom in congenital syphilis, with a prevalence exceeding 50% [31]. IK frequently occurs with the Hutchinson triad, which includes keratitis, deafness, and Hutchinson’s teeth [32].

Recent advances in imaging technologies have provided greater clarity regarding the clinical features of IK. Optical coherence tomography (OCT) revealed that about 45% of patients with syphilitic IK showed pyramidal hyperreflective foci in the outer retina and retinal pigment epithelium (RPE). In contrast, typical placoid lesions on fundus examination were absent in 54% of patients, suggesting that OCT can detect minor retinal changes not readily apparent during clinical evaluation. The lesion disappeared in 68% of patients after treatment. The visual acuity of patients also significantly improved, further supporting that syphilitic IK is curable [33] (Table 1).

### 2.2. Posterior Segment Manifestation

Posterior uveitis and panuveitis: Syphilitic uveitis is the most common ocular manifestation among patients with syphilis and is particularly common in the posterior segment of the eye. A study that included 388 syphilis patients showed that uveitis is the most common ocular manifestation (45.6%), followed by retinitis (12.7%) and optic neuritis (11.4%) [11]. Zhang et al. reported that posterior segment involvement was observed in 97.0% of eyes (32 out of 33) with ocular syphilis in northern China [17]. A retrospective multicenter study conducted in the United States by Oliver et al. demonstrated that hazy vision, foveal involvement, and posterior uveitis were the predominant clinical findings [11]. Although syphilitic uveitis can occur at any stage of the disease, it is most commonly reported during the late latent and tertiary stages. The presence of uveitis at these stages often indicates underlying immune dysfunction [35,36].

Overall, posterior segment manifestations have intricate and varied clinical manifestations. Precise diagnosis depends on a strong clinical suspicion, systematic imaging (OCT and fluorescein angiography), and, when warranted, evaluation of serum and cerebrospinal fluid. Timely diagnosis and treatment are crucial to preserve visual function.

Retinal Vasculitis: Retinal vasculitis is a significant, although sometimes neglected, manifestation. Jabbehdari et al. observed that it may occur independently or in conjunction with vitritis and choroiditis and is frequently mistaken clinically for leukoaraiosis or tuberculous uveitis [37]. In a prospective cohort study conducted in Saudi Arabia, Alhawsawi et al. found that vitritis was observed in 36.3% of affected eyes, while posterior uveitis was detected in 22.4% of eyes, with some individuals exhibiting optic nerve papillary edema [15].

Retinal Detachment: Although rare, ocular syphilis can cause retinal detachment, including exudative and rhegmatogenous retinal detachment. Syphilitic retinal detachment is often accompanied by uveitis, retinitis, and vitritis [38,39]. In most cases of exudative retinal detachment, patients experience substantial resolution after intravenous penicillin therapy, while a surgical approach is often required in cases of rhegmatogenous retinal detachment, and the visual acuity of these patients remains guarded [40].

Syphilitic Optic Neuropathies: Optic neuritis is a rare ocular complication among patients with syphilis. A study including 577 patients with syphilis showed that 4.3% presented with optic disc edema and 1.2%  with optic atrophy [38]. Patients may exhibit unilateral or bilateral perineuritis, anterior or retrobulbar optic neuritis, or papilledema [41]. Distinguishing syphilitic optic neuritis from other forms of noninfectious optic neuritis presents significant challenges. The peripapillary retinal nerve fiber layer (pRNFL) was used as a marker for optic neuropathies and some systemic pathologies [42]; however, this association was absent in syphilitic optic neuropathies [38], suggesting that syphilitic optic neuropathies represent an episodic response to optic nerve or cerebral infection rather than a chronic process.

### 2.3. ASPPC

**Figure 1:**
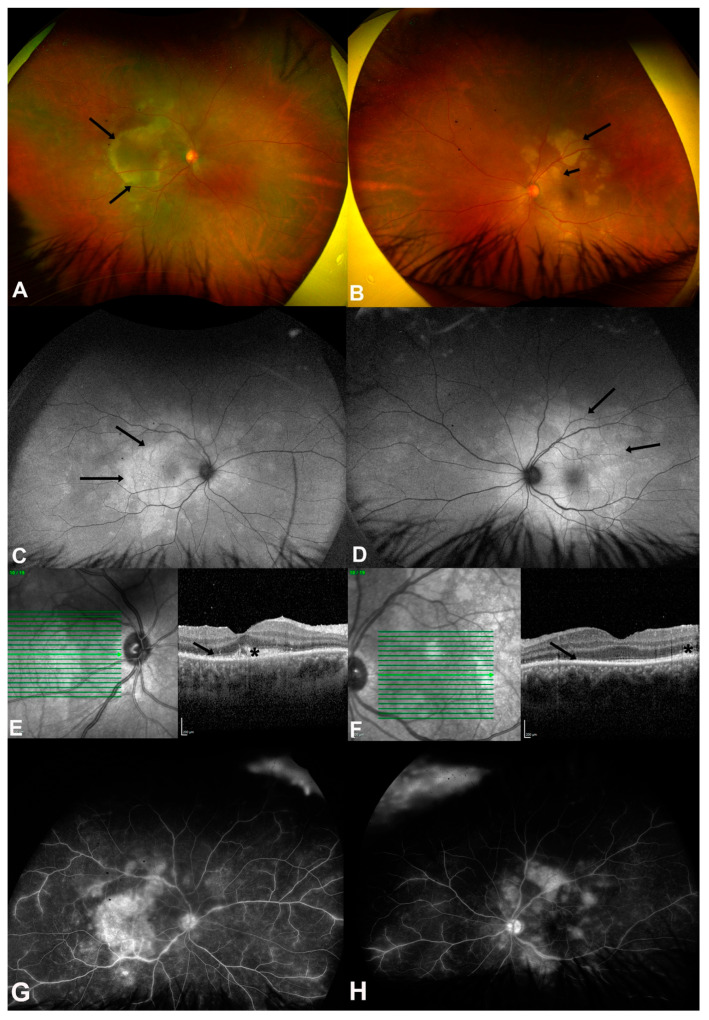
(**A**,**B**) are color fundus photos of both eyes of a 25-year-old male (case 4) showing multiple posterior placoid lesions at the macula (black arrows) and posterior pole, indicating acute syphilitic posterior placoid chorioretinopathy (ASPPC). (**C**,**D**) are fundus autofluorescence (FAF) photos showing hyperautofluorescent areas more extensive than the lesions that were found clinically (black arrows). (**E**,**F**) are the spectral domain optical coherence tomography (SD-OCT) of both eyes showing disrupted ellipsoid zones (black arrows), nodular elevations (asterisks) of retinal pigment epithelium (RPE), and intraretinal hyperreflective lesions. (**G**,**H**) are fundus fluorescein angiography (FFA) photos showing hyperfluorescent discs, late staining of the placoid lesions, and vascular leakage at the posterior pole in both eyes. (Image source: Magliyah, M., Al-Dhibi, H., Alsulaiman, S., Albloushi, A. F., & AlHawsawi, A. (2025). The Clinical Spectrum and Outcomes of Ocular Syphilis Disease in Saudi Arabia: The Emergence of a Uveitic Masquerader. Investigative Ophthalmology & Visual Science, 66(8), 5455–5455 [15]. This image is used under the Creative Commons Attribution-NonCommercial-NoDerivatives 4.0 International License (CC BY-NC-ND 4.0). No modifications were made. License: https://creativecommons.org/licenses/by-nc-nd/4.0/ (accessed on 18 July 2025)).

Acute syphilitic posterior placoid chorioretinitis (ASPPC) is a well-recognized manifestation of syphilitic involvement in the posterior segment, clinically characterized by discoid yellow lesions in the macula and the degradation of outer retinal components [36]. Retinopathy in ASPPC frequently occurs alongside varying levels of vitreous inflammation and may manifest as superficial hemorrhages, retinal vasculitis, optic disc edema, and retinal pigment epithelium (RPE) detachment [43]. Clinical examination and multimodal imaging have demonstrated that both the outer retina and choroidal capillaries are involved in ASPPC. Notably, choroidal capillary dropout has been documented [44], and this layer is considered the principal anatomical site of inflammation [45]. In a comprehensive study that included 387 eyes of individuals with ASPPC, only two cases of choroidal neovascular membrane (CNV) formation were reported. This low incidence, combined with the overall restoration of anatomical integrity following appropriate treatment, suggested that Bruch’s membrane is generally resistant to damage in ASPPC. Moreover, although ASPPC demonstrates a higher rate of serological titer levels (especially RPR) compared to other subtypes, it is less frequently accompanied by optic nerve involvement. Routine RPR analysis is not considered a standard diagnostic step, as it rarely alters treatment decisions [46] (Figure 1).

### 2.4. Neuro-Ophthalmic Complications

Syphilitic neuro-ophthalmological manifestations are considered a form of neurosyphilis. Common presentations include optic neuritis, papilledema, neuroretinitis, and cranial nerve palsies [47,48]. According to Garg et al., syphilitic optic neuropathy typically presents as acute and bilateral, with magnetic resonance imaging (MRI) and CSF analysis serving as valuable tools for differential diagnosis [49]. Chauhan et al. further emphasized that optic nerve involvement is a common finding in patients with HIV-associated syphilis, suggesting a link between immunosuppression and increased vulnerability to optic nerve damage [50]. Sivabalan et al. reported a case of neurosyphilis presenting with diplopia and motor cranial nerve palsy, which was ultimately diagnosed through CSF analysis [51]. Similarly, Levy et al. demonstrated that syphilitic optic neuropathy may be the sole presenting feature of neurosyphilis in patients co-infected with HIV, underscoring the critical role of CSF examination in early detection [52] (Table 2).

## 3. Diagnostic Challenges and Advanced Modalities

As its other name, “the great imitator,” indicates, the manifestation of ocular syphilis varies and is nonspecific, posing significant diagnostic challenges [53,54]. Early diagnosis and prompt treatment of syphilitic uveitis are critical for controlling infection and preserving visual function [55,56]. O’Connell et al. reported that treatment for syphilitic panuveitis at an early stage—particularly in the absence of systemic manifestations—significantly improves visual outcomes. Their findings also highlight the importance of timely serological confirmation and the rapid initiation of antibiotic therapy for a favorable visual prognosis [57].

### 3.1. Limitations of Traditional Serology Test in Ocular Syphilis

Although classic serological tests, such as non-treponemal tests (NTTs) and treponemal tests (TTs), remain in use for diagnosing systemic syphilis, their diagnostic efficacy in ocular syphilis is increasingly under scrutiny [58]. In the conventional approach, NTTs, such as RPR or VDRL, were used for initial screening, followed by TTs for confirmation. However, these approaches may show false-negative or “seronegative” results in some individuals with neurosyphilis. Conventional serological testing methods like RPR or VDRL exhibit a sensitivity range of 48.7–76.1% (with some studies indicating lower values) when compared with dark-field microscopy, the gold standard for diagnosing primary syphilis in its initial stages. The sensitivity of VDRL ranges from 50.0% to 78.4%, with some investigations indicating lower values [59,60]. The reduced sensitivity indicates that a considerable number of patients with early-stage syphilis may exhibit a “false-negative,” postponing treatment.

This phenomenon is extremely important in ocular syphilis. Mohareb et al. demonstrated that around 40% of patients with ocular syphilis exhibited a nonreactive or low-titer RPR, with these patients sharing characteristics similar to those with high-titer RPR. This highlights the limitations of conventional algorithms in identifying ocular syphilis. They emphasized that these traditional algorithms may overlook latent or advanced cases of ocular syphilis [56]. Furthermore, Wills et al. demonstrated that the specificity and sensitivity of serological tests are inadequate for neurological subtypes, including ocular syphilis [61]. Research indicates that TT may yield lifelong positivity, and the combination of TT and NTT may prove ineffective in detecting infections in patients with solely ocular manifestations and atypical systemic symptoms [62,63]. The above findings highlight the need for adjunctive diagnostic strategies such as anterior chamber aqueous humor PCR and localized intraocular antibody assays to improve diagnostic accuracy.

### 3.2. Cerebrospinal Fluid Antibody Analysis

Cerebrospinal fluid (CSF) antibody testing is useful for diagnosing and treating ocular syphilis, especially in immunocompromised people or those who might have neurological involvement [64]. The 2010 Centers for Disease Control and Prevention (CDC) Sexually Transmitted Disease (STD) Treatment Guidelines state that the CSF FTA-ABS test is less specific for neurosyphilis than the CSF-VDRL but is highly sensitive [65]. Importantly, while a negative CSF treponemal test makes the diagnosis of neurosyphilis unlikely, such assays are not recommended for monitoring treatment response [66]. Instead, therapeutic monitoring relies on serum non-treponemal tests, such as the rapid plasma reagin RPR or VDRL test, with a ≥4-fold decline in titer regarded as an indicator of treatment success [67]. Therefore, all guidance regarding treatment follow-up should focus on serial non-treponemal titers rather than CSF antibody assays.

### 3.3. Imaging-Driven Diagnosis

Recent advancements in ocular imaging technology have transformed clinical evaluation by enabling earlier and more precise diagnosis of ocular syphilis. Swept-source anterior segment optical coherence tomography (SS-ASOCT) allows for the detailed observation of iris abnormalities that are often difficult to detect using traditional slit-lamp examination. This new technology is valuable for identifying atypical presentations of ocular syphilis manifestations [68]. Near-infrared reflectance (NIR) imaging has demonstrated considerable efficacy in identifying acute syphilitic outer retinitis. Chen et al. demonstrated that NIR imaging can detect hyperreflective lesions associated with inflammation, indicating active disease even in cases with inconclusive conventional serological results. Another study that included 39 eyes with ocular syphilis showed that NIR imaging can highlight subtle retinal changes, including changes in the ellipsoid zone layer and subretinal deposits [69]. These findings suggest that NIR imaging may serve as a valuable adjunct in the early detection of ocular syphilis.

**Figure 2:**
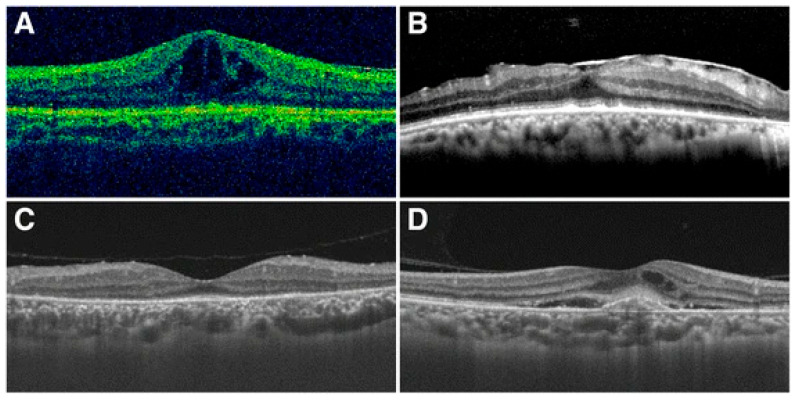
Typical pictures of macular changes. (**A**) cystoid macular edema; (**B**) epiretinal membrane; (**C**) disappearance of the photoreceptor inner segment–outer segment junction line; (**D**) choroidal neovascularization (image source: Zhang, X., Du, Q., Ma, F., Lu, Y., Wang, M., & Li, X. (2017). Characteristics of syphilitic uveitis in northern China. BMC Ophthalmology, 17(1), 95 [17]. This image is used under the Creative Commons Attribution 4.0 International License (CC BY 4.0), which permits use, sharing, adaptation, distribution and reproduction in any medium or format, as long as appropriate credit is given to the original author(s) and the source. License: https://creativecommons.org/licenses/by/4.0/ (accessed on 20 July 2025)).

SD-OCT identifies inflammatory alterations in the vitreoretinal interface, retina, and choroid [43]. A case series involving four patients with syphilitic optic neuropathy revealed that disruptions in the ellipsoid zone and/or placoid chorioretinitis, characterized by retinal pigment epithelium excrescences, were noted in macular optical coherence tomography [17] (Figure 2). Therefore, clinicians are advised to acquire macular OCT for all patients exhibiting undifferentiated optic neuropathy [70].

### 3.4. Molecular Diagnostics

Polymerase chain reaction (PCR) of aqueous or vitreous fluid is currently one of the most reliable ways to detect the DNA of syphilis spirochete. Méndez-Rodríguez et al. showed that PCR results were specific even in cases with atypical clinical presentations or negative serological results [71]. A study that reviewed the syphilis diagnostic literature of the past 50 years indicated a sensitivity exceeding 85% for detecting ocular syphilis during the active inflammatory phase and was especially helpful for people who were co-infected with HIV [72]. Cerebrospinal fluid PCR can also help tell the difference between neurosyphilis and simple ocular involvement when optic neuropathy is suspected, even though it is not utilized as often. Real-time quantitative PCR (qPCR), in conjunction with conventional PCR, has been employed to quantify bacterial load and assess treatment efficacy. Sena et al. demonstrated that qPCR can not only detect *Treponema pallidum* in ocular tissues but also monitor the therapeutic response to antibiotic treatment [73].

Despite certain limitations, such as the need for invasive sample collection and the requirement for specialized laboratory facilities, molecular diagnostic methods remain valuable for identifying the precise etiology of ocular syphilis, especially when used in conjunction with serological testing and ocular imaging. Recent diagnostic algorithms recommend an integrated approach combining molecular techniques (e.g., PCR), imaging modalities (such as OCT or fluorescein angiography), and clinical red flags, such as unexplained acute posterior uveitis. This strategy is especially critical for immunocompromised patients, in whom antibody production may be impaired due to immunosuppressive therapies, such as CD20 monoclonal antibodies [74] (Table 3).

## 4. Therapeutic Innovations in Ocular Syphilis

### 4.1. Adjunctive and Alternative Strategies

Although intravenous penicillin G is the primary treatment for ocular syphilis [75], treatment may not always proceed smoothly. In real-world clinical settings, coordinating outpatient intravenous penicillin therapy can be particularly challenging, especially for people who inject drugs (PWIDs) and those lacking stable housing or adequate social support [75,76]. In addition, there is a growing clinical investigation into supplementary and alternative therapeutic approaches in instances of penicillin allergy, treatment failure, or co-infection.

Ceftriaxone (2 g daily for 10–14 days) has been proposed as a viable alternative for patients with penicillin allergies, demonstrating favorable central nervous system penetration. Workowski et al. assert that, although ceftriaxone demonstrates in vitro action against syphilis spirochetes, rigorous evidence supporting its clinical usefulness in ocular syphilis is lacking [75,77]. In resource-limited regions, oral doxycycline (100 mg bi-daily for 28 days) is occasionally employed as a pragmatic substitute. Doxycycline should be utilized exclusively when penicillin and ceftriaxone are unavailable [78]. Importantly, evidence for doxycycline in ocular and neurosyphilis remains very limited, and it should not be regarded as equivalent to penicillin therapy [79]. As co-infection with HIV compromises the integrity of the blood–brain barrier, preventing doxycycline from attaining effective concentrations in the cerebrospinal fluid (CSF) (<0.5 μg/mL, whereas the minimum inhibitory concentration (MIC) for syphilis spirochetes is 0.1 μg/mL), adjunctive corticosteroids may mitigate inflammatory consequences (e.g., uveitis); however, their application remains empirical [80].

Moreover, intravitreal antibiotic injections and immunomodulatory therapy have been employed in certain refractory instances; however, these approaches remain experimental and lack validation from extensive investigations. In addition, patients with compromised immune function may exhibit suboptimal responses to standard treatment. Prospective cohort studies involving patients with HIV-associated neurosyphilis have identified CSF CXCL13 levels as a reliable biomarker for active neurosyphilis and a potential tool for monitoring treatment response. In some cases, elevated CSF CXCL13 levels persist even after the completion of standard penicillin therapy, suggesting the incomplete clearance of the pathogen and the possible need for retreatment [81]. This means that high-risk individuals need to be closely monitored after therapy, which may include checking serological markers and performing a lumbar puncture if needed (Table 4).

### 4.2. Vaccine Development

Currently, there is no available vaccine for ocular syphilis or systemic *Treponema pallidum* infection, despite substantial efforts in recent years to develop an effective syphilis vaccine. The pathogen’s ability to evade the host immune response, along with the limited antigenic targets due to its sparse outer membrane structure, has long posed significant challenges to vaccine development [82]. T. pallidum expresses multiple surface-accessible proteins that play key roles in endothelial transmigration. A recent study has shown that these antigens can induce strong bactericidal antibody responses, offering promising targets for future syphilis vaccine development [83]. Also, DNA vaccines that contain T. pallidum antigens (such as Tp0751) have helped to prevent dissemination to the eyes and central nervous system in the rabbit model, which is the gold standard for syphilis research. Even though full immunological protection has not been reached yet, the partial protective effect seen in animal models gives rise to hope that we can lessen the difficulties of ocular syphilis [84].

Ocular syphilis has often been classified as a neurological manifestation due to its involvement of immune-privileged sites, such as the eye and central nervous system. Therefore, future vaccine strategies should be designed to enhance both systemic and mucosal immunity in order to prevent pathogen dissemination to these protected parts. In addition, effective vaccines should elicit durable T-cell-mediated immune responses capable of conferring protection against ocular and neurologic involvement. Ultimately, vaccination should aim not only to prevent systemic infection but also to reduce the risk of vision-threatening ocular syphilis [85].

## 5. Co-Infection with Retrovirus: Synergistic Pathogenesis and Management

### 5.1. HIV Co-Infection in Syphilitic Patients

A close association between HIV and syphilis has been well established, as the two infections share common risk factors—most notably sexual transmission, particularly among MSM. Syphilis patients co-infected with HIV were nearly twice as likely to report ocular symptoms as were patients without HIV [43]. In a study conducted in South Africa involving 215 eyes with ocular syphilis, HIV co-infection was identified in as many as 52.1% of patients [86]. Moreover, HIV co-infection in patients with syphilis poses additional challenges for diagnosis and treatment, often complicating their clinical management [87]. Amaratunge et al. demonstrated that co-infection significantly modifies the clinical phenotype of ocular syphilis. In comparison to HIV-negative patients, individuals co-infected with HIV exhibited a higher likelihood of developing posterior uveitis or panuveitis [88]. Ahmed et al. provided more detailed results based on HIV status. They found that posterior uveitis and optic neuropathy were more common in HIV-positive patients, while anterior uveitis was more common in HIV-negative patients. This demonstrates how the immune system can influence disease presentation [89]. However, some studies reached different conclusions. A systematic review encompassing 95 studies indicated that HIV status, CD4 cell count, and HIV viral load did not significantly influence visual acuity outcomes in eyes affected by ocular syphilis [90]. Moreover, HIV co-infection changes the typical serological response and negatively impacts treatment outcomes in patients with syphilis. CSF abnormalities, indicative of neurosyphilis, were more frequently observed in HIV-positive individuals, 83% of whom underwent lumbar puncture compared to 61% of HIV-negative patients [87,88].

HIV co-infection also modifies the host inflammatory response to *Treponema pallidum*, thereby necessitating individualized or tailored therapeutic strategies. A systematic review by Wu et al. indicated that the prognosis for visual recovery in ocular syphilis patients co-infected with HIV is generally poorer compared to HIV-negative individuals [90] (Table 5).

### 5.2. HTLV-1 Co-Infection in Syphilitic Patients

To date, studies specifically focused on the relationship between HTLV-1 and syphilis remain limited. However, several reports have highlighted a potential association. A study from Brazil found that 23% of patients with HTLV-1 also had syphilis, a rate comparable to that observed in HIV-infected individuals (26%) [91]. Another study from Spain, involving 2524 patients with STIs, reported an overall HTLV prevalence of 0.5%, with HTLV-1 accounting for 0.2% [92]. The observed prevalence is markedly greater than that found in the general population, indicating a substantial yet underappreciated association between STIs and HTLV-1.

In fact, the WHO now recognizes HTLV-1 as a sexually transmitted infection [93]. This may explain why HTLV-1 is more frequently observed among patients with syphilis and implies that its prevalence in this population is likely underestimated. In Japan, the implementation of antenatal HTLV-1 screening is anticipated to result in horizontal transmission becoming the predominant mode of infection among adolescents and young adults. The incidence of HTLV-1 seroconversions is reported as 1.54 per 100,000 person-years for men and 4.21 for women [94]. This trend is particularly relevant to ocular disease, as cases have been documented in which HTLV-1 infection acquired through sexual contact led to ocular manifestations in young individuals—even in the presence of low proviral load and short latency [95,96].

Given these findings, greater attention should be paid to HTLV-1 screening in syphilis patients, particularly those presenting with unexplained ocular inflammation. Diagnosing ocular syphilis in HTLV-1 endemic regions necessitates heightened awareness and tailored therapeutic techniques alongside immune function assessment.

## 6. Global Health Strategies for Ocular Syphilis

Despite its potential to cause irreversible blindness, ocular syphilis is often overlooked in global STI surveillance programs. To improve control efforts, it should be incorporated into broader STI programs, with a particular focus on equitable access to diagnostics and treatment. The World Health Organization (WHO) has called for expanded syphilis testing and treatment access, especially in low- and middle-income countries (LMICs)—particularly for patients presenting with atypical symptoms, including ocular manifestations [2]. Including ocular syphilis as a distinct category within global STI data platforms would enhance visibility and guide resource allocation.

Models like the Minnesota One Health Antibiotic Stewardship Collaborative (MOHASC), although not specific to ocular syphilis, demonstrate the value of interdisciplinary, cross-sector collaboration in antimicrobial stewardship. Such frameworks can inform integrated strategies for STI management, emphasizing surveillance, education, and responsible antibiotic use [97].

To close the diagnostic gap, especially in resource-limited settings, investment in molecular diagnostic infrastructure and frontline healthcare training is essential. The WHO Global STI Strategy (2022–2030), which targets the elimination of congenital and adult syphilis, also indirectly supports the early detection and management of ocular syphilis [98]. In addition, to strengthen program monitoring, the following sentinel indicators are recommended: the median time-to-treatment from the onset of ocular symptoms—since earlier antibiotic intervention has been shown to improve visual recovery trajectories [99]; and (ii) the percentage of affected eyes achieving visual acuity of ≥20/40 at 3–6 months post-treatment—a meaningful measure of functional outcome, with studies reporting favorable long-term VA recovery around this threshold [7,9]. Moving forward, equitable funding, cross-border collaboration, and the use of digital health tools will be crucial in promoting accessible and effective ocular syphilis care worldwide.

## Figures and Tables

**Table 1:** Anterior segment phenotypes of ocular syphilis: key signs, pitfalls, differentials, imaging correlates, and evolution after treatment.

Study	Clinical Entity	Key Signs	Common Pitfalls	Key Differentials	Imaging Correlates	Evolution After Treatment
Alhawsawi et al. [15]	Anterior uveitis	Blurred vision, floaters, red eye with pain, conjunctival injection	Misdiagnosed as autoimmune uveitis	HLA-B27 anterior uveitis and viral uveitis	Slit-lamp; OCT	Responsive to IV penicillin; VA recovery varies
Shahid et al. [22]	Anterior uveitis	Similar prevalence to posterior uveitis; 25.9% anterior vs. 22.4% posterior	Mistaken for idiopathic cases	Idiopathic uveitis and sarcoidosis	OCT; FA	Improves with systemic antibiotics
Cillino et al. [25]	Eyelid chancre with HIV co-infection	Painless eyelid ulcer, resolving lesion at lateral canthus → later bilateral chorioretinitis	Misdiagnosed as chalazion	Viral keratitis and chalazion	Fundus photo; FA	Lesions resolved after penicillin
Zhu et al. [34]	Syphilitic scleritis	Painful red eye, nodular anterior scleritis, necrotizing form is rare	Misdiagnosed as rheumatoid scleritis	RA, TB scleritis, HSV	Anterior segment OCT	Responds to IV penicillin; superficial type; good prognosis
Knox et al. [32]	Interstitial keratitis (congenital syphilis)	Keratitis, deafness, Hutchinson’s teeth (Hutchinson’s triad)	Misdiagnosed as herpetic keratitis	HSV keratitis and autoimmune IK	OCT: stromal haze and outer retina changes)	Response to antibiotics; vision recovery possible

**Table 2:** Posterior segment phenotypes of ocular syphilis: key signs, diagnostic pitfalls, differentials, imaging correlates, and post-treatment evolution.

Study	Clinical Entity	Key Signs	Common Pitfalls	Key Differentials	Imaging Correlates	Evolution After Treatment
Oliver et al. (2016) [11]; Zhang et al. (2017) [17]	Posterior uveitis/panuveitis	Blurred vision, foveal involvement, vitreous haze	Misdiagnosed as autoimmune posterior uveitis	Tuberculous uveitis; sarcoid uveitis	OCT/FA: vitritis and chorioretinal lesions	Responds to IV penicillin, with partial visual acuity recovery
Jabbehdari et al. (2017) [37]; Alhawsawi et al. (2025) [15]	Retinal vasculitis	Perivascular sheathing and vascular leakage on FA	Mistaken for TB or idiopathic vasculitis	Behçet’s disease; TB uveitis	FA: vascular leakage; OCT: retinal edema	Improves with systemic antibiotics ± corticosteroids
Du et al.(2025) [38]; JUMPER et al.(2000) [39]; Shughoury et al.(2024) [40]	Retinal detachment	Exudative or rhegmatogenous detachment, often with uveitis/retinitis	Misdiagnosed as autoimmune retinopathy	Vogt–Koyanagi–Harada disease; CMV retinitis	OCT: subretinal fluid; FA: pooling	Exudative detachment resolves with penicillin; RRD often needs surgery
Du et al.(2025) [38]; Smith et al.(2006) [41]; Moore et al.(2015) [42]	Optic neuropathies	Optic neuritis, disc edema, papilledema	Misdiagnosed as demyelinating optic neuritis	Multiple sclerosis, NMO, ischemic optic neuropathy	OCT: RNFL changes; MRI: optic nerve enhancement	Variable recovery; poorer outcomes in HIV+
Eandi et al.(2012) [36]; Pichi et al.(2014) [43]; Wai et al.(2022) [44]; Herbort et al.(2020) [45]	ASPPC	Macular placoid yellow lesions, RPE disruption, vitreous inflammation	Mistaken for APMPPE or serpiginous choroiditis	APMPPE; MEWDS	OCT: EZ loss and RPE nodules; FAF: hyperautofluorescence; FA/ICGA: choroidal hypoperfusion	Usually resolves with antibiotics; anatomical recovery common

**Table 3:** Diagnostic modalities in ocular and neurosyphilis: sensitivity/specificity ranges, key findings, and limitations.

Modality	Sensitivity/Specificity (Reported Ranges)	Key Limitations	References
Non-treponemal tests (NTT: RPR and VDRL)	Sensitivity 48.7–76.1% vs. dark-field microscopy; CSF-VDRL sensitivity 50–78.4%	False-negative results (esp. ocular/neuro involvement); up to 40% of ocular syphilis patients with low/negative RPR; poor sensitivity for neurosyphilis	[56,58,59,60,61]
Treponemal tests (TT: TPHA and FTA-ABS)	High sensitivity; lifelong positivity	Cannot distinguish active vs. past infection; not reliable for follow-up or isolated ocular disease	[58,62,63]
CSF-VDRL	Highly specific but insensitive	Negative result does not exclude neurosyphilis; invasive	[64,65]
CSF-FTA-ABS	Highly sensitive, less specific than CSF-VDRL	Not recommended for monitoring treatment; may yield false positives	[64,65,66]
Intraocular antibody index	Adjunctive diagnostic tool (no standardized sensitivity)	Limited validation; may be negative in immunocompromised patients	[56,64]
Aqueous/vitreous PCR (*Treponema pallidum* DNA)	Sensitivity > 85% in active phase; high specificity; qPCR can monitor bacterial load	Requires invasive sample; limited lab availability	[71,72,73]
OCT (SD-OCT and SS-ASOCT)	Detects retinal/choroidal inflammatory changes; macular OCT valuable in optic neuropathy	Nonspecific; cannot directly confirm syphilis	[17,43,68,70]
Near-infrared reflectance (NIR)	Sensitive to syphilitic outer retinitis, detects subtle retinal lesions	Adjunctive only; not specific	[68,69]
Fundus autofluorescence (FAF) and OCT angiography (OCTA)	Described as adjunctive, useful for structural/vascular changes	Lack of standardized sensitivity data	[68,70]

**Table 4:** Treatment regimens for ocular/neurosyphilis: regimen, dose, duration, advantages, disadvantages, evidence, and considerations in special situations.

Regimen	Dose and Duration	Pros/Cons	Evidence Level	Special Situations
IV Penicillin G	18–24 million units/day (3–4 million units q4h or continuous infusion), 10–14 days	Gold standard; excellent CNS penetration; proven efficacy. Limited by need for hospitalization or IV access.	High (CDC guidelines, RCTs, cohort studies)	First-line for all patients, including pregnancy and HIV co-infection [75,77].
Ceftriaxone	2 g IV/IM daily, 10–14 days	Alternative in penicillin allergy; good CNS penetration. Limited evidence in ocular syphilis; efficacy not fully established.	Moderate (observational studies and case reports)	Consider only if penicillin cannot be given. Use with caution in HIV [75,77].
Doxycycline	100 mg orally twice daily, 28 days	Oral route convenient; accessible in resource-limited areas. Poor CSF penetration; limited evidence in ocular/neurosyphilis. Not equivalent to penicillin.	Low (small case series and expert opinion)	Not recommended except when penicillin/ceftriaxone unavailable. Contraindicated in pregnancy; limited efficacy in HIV [78,79].
Adjunctive Corticosteroids	Prednisone (short taper course; variable dose)	May reduce inflammation and Jarisch–Herxheimer reaction; evidence is empirical. No effect on pathogen clearance.	Low (case series and expert consensus)	Used cautiously to control uveitis or severe inflammation [80].
Experimental/Emerging Approaches	Intravitreal antibiotics; immunomodulators; biomarker-guided retreatment (e.g., CSF CXCL13)	Potential for refractory cases; biomarkers may help monitor treatment. Not validated by large trials.	Very low (pilot studies and animal models)	Consider in HIV-associated neurosyphilis or retreatment contexts [81].

**Table 5:** Clinical phenotype and outcomes of ocular/neurosyphilis according to HIV status.

Feature/Outcome	HIV-Negative Patients	HIV-Positive Patients	References
Frequency of ocular involvement	Lower frequency of ocular symptoms in syphilis patients	Nearly 2× higher risk of ocular symptoms among syphilis patients with HIV	[43,86]
Common ocular phenotype	More anterior/intermediate uveitis; focal retinitis; placoid chorioretinitis	Higher frequency of posterior uveitis, panuveitis, and optic neuropathy	[87,88,89]
CSF abnormalities	Less frequent; lumbar puncture performed in ~61%	More frequent (pleocytosis and protein elevation); lumbar puncture performed in ~83%	[87,88]
Serological response	Conventional NTT/TT more reliable, but false negatives occur	Serological response altered; atypical or discordant serology under HIV-induced immune dysregulation	[56,62,63,87]
Treatment response	Generally favorable with IV penicillin	Suboptimal response; higher risk of incomplete clearance; possible need for retreatment; CSF CXCL13 may persist	[75,81,87,88]
Visual prognosis	Good if treated early; favorable outcomes in many cases	Prognosis often poorer; systematic reviews show higher recurrence and worse visual recovery	[57,90]

## Data Availability

No new data were created or analyzed in this study. Data sharing is not applicable to this article.
